# Detection of COVID-19 Using Deep Learning Techniques and Cost Effectiveness Evaluation: A Survey

**DOI:** 10.3389/frai.2022.912022

**Published:** 2022-05-27

**Authors:** Manoj Kumar M. V., Shadi Atalla, Nasser Almuraqab, Immanuel Azaad Moonesar

**Affiliations:** ^1^Department of Information Science and Engineering, Nitte Meenakshi Institute of Technology, Bangalore, India; ^2^College of Engineering & Information Technology, University of Dubai, Dubai, United Arab Emirates; ^3^Dubai Business School, University of Dubai, Dubai, United Arab Emirates; ^4^Health Adminstration & Policy – Academic Affairs, Mohammed Bin Rashid School of Government (MBRSG), Dubai, United Arab Emirates

**Keywords:** COVID-19 diagnosis, deep learning, artificial intelligence, chest X-ray, chest CT, COVID financial management, insurance

## Abstract

Graphical-design-based symptomatic techniques in pandemics perform a quintessential purpose in screening hit causes that comparatively render better outcomes amongst the principal radioscopy mechanisms in recognizing and diagnosing COVID-19 cases. The deep learning paradigm has been applied vastly to investigate radiographic images such as Chest X-Rays (CXR) and CT scan images. These radiographic images are rich in information such as patterns and clusters like structures, which are evident in conformance and detection of COVID-19 like pandemics. This paper aims to comprehensively study and analyze detection methodology based on Deep learning techniques for COVID-19 diagnosis. Deep learning technology is a good, practical, and affordable modality that can be deemed a reliable technique for adequately diagnosing the COVID-19 virus. Furthermore, the research determines the potential to enhance image character through artificial intelligence and distinguishes the most inexpensive and most trustworthy imaging method to anticipate dreadful viruses. This paper further discusses the cost-effectiveness of the surveyed methods for detecting COVID-19, in contrast with the other methods. Several finance-related aspects of COVID-19 detection effectiveness of different methods used for COVID-19 detection have been discussed. Overall, this study presents an overview of COVID-19 detection using deep learning methods and their cost-effectiveness and financial implications from the perspective of insurance claim settlement.

## Introduction

Coronavirus is a zoonotic virus and an RNA virus in the family Coronaviridae, wherein these zoonotic diseases are infectious diseases caused by the transmission of pathogens from animals to humans. It is a class of viruses that belong to respiratory contagion. The virus is entitled Coronavirus because of the crown-like spikes on the virus's outer covering. Corona is a sort of infection that creates respiratory ailments in humans (Darapaneni et al., 2020).

The various deadly coronaviruses discovered to date are,

1. *SARS-CoV* induces s*evere acute respiratory syndrome (SARS)*
2. *MERS-CoV* which causes *Middle East respiratory syndrome (MERS)*
3. *SARS-CoV2* which is novel coronavirus that causes *COVID-19*.

Novel Coronavirus (COVID-19) originated in bats and was transmitted to humans in December 2019 by an unknown animal in Wuhan, China (El Gannour et al., 2020). People who feel high temperatures, coughing, aching throats, tiredness, muscle aches, throat pain, and difficulty breathing could be affected by this virus (Thepade and Jadhav, 2020). The World Health Organization declared COVID-19 a Public Health Emergency of International Concern on January 30, 2020, and it was declared a global pandemic on March 11, 2020.

**Figure 1:**
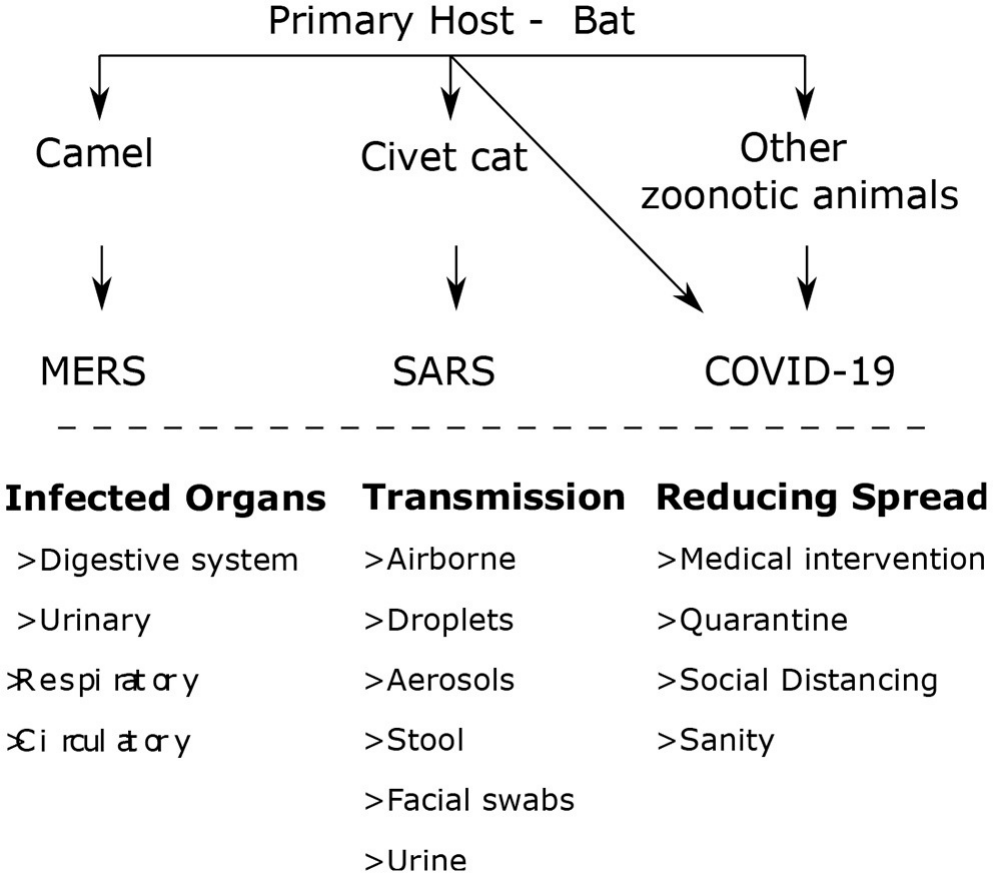
Transmission of the SARS-COV (Darapaneni et al., 2020).

Figure 1 demonstrates the transmission of the severe acute respiratory syndrome coronavirus (SARS-CoV). SARS-CoV-2 strains are classified into four genera: alpha, beta, gamma, and delta. However, the genera alpha and beta descended from bats, whereas gamma and delta descended from birds and peccary gene provision. For SARS and MERS, civet cats and camels act as the proprietors. The most common symptom associated with the COVID-19 dreadful disease is Pneumonia, which appears to be the most common indication of contamination, along with other primary symptoms such as fever, cold, body pain, sore throat, and epistaxis (Darapaneni et al., 2020).

The total number of cases in the first wave till June 2020 real cases are 8,708,008, while the inclusive estimate of mortality is 461,715 (WHO Statistics). However, the severity of COVID-19 demonstrates the second wave total number of instances till June 1, 2021. As a result, the absolute number of verified cases is 171,782,908, while the cumulative mortality estimate is 3,698,621^1^,^2^

**Figure 2:**
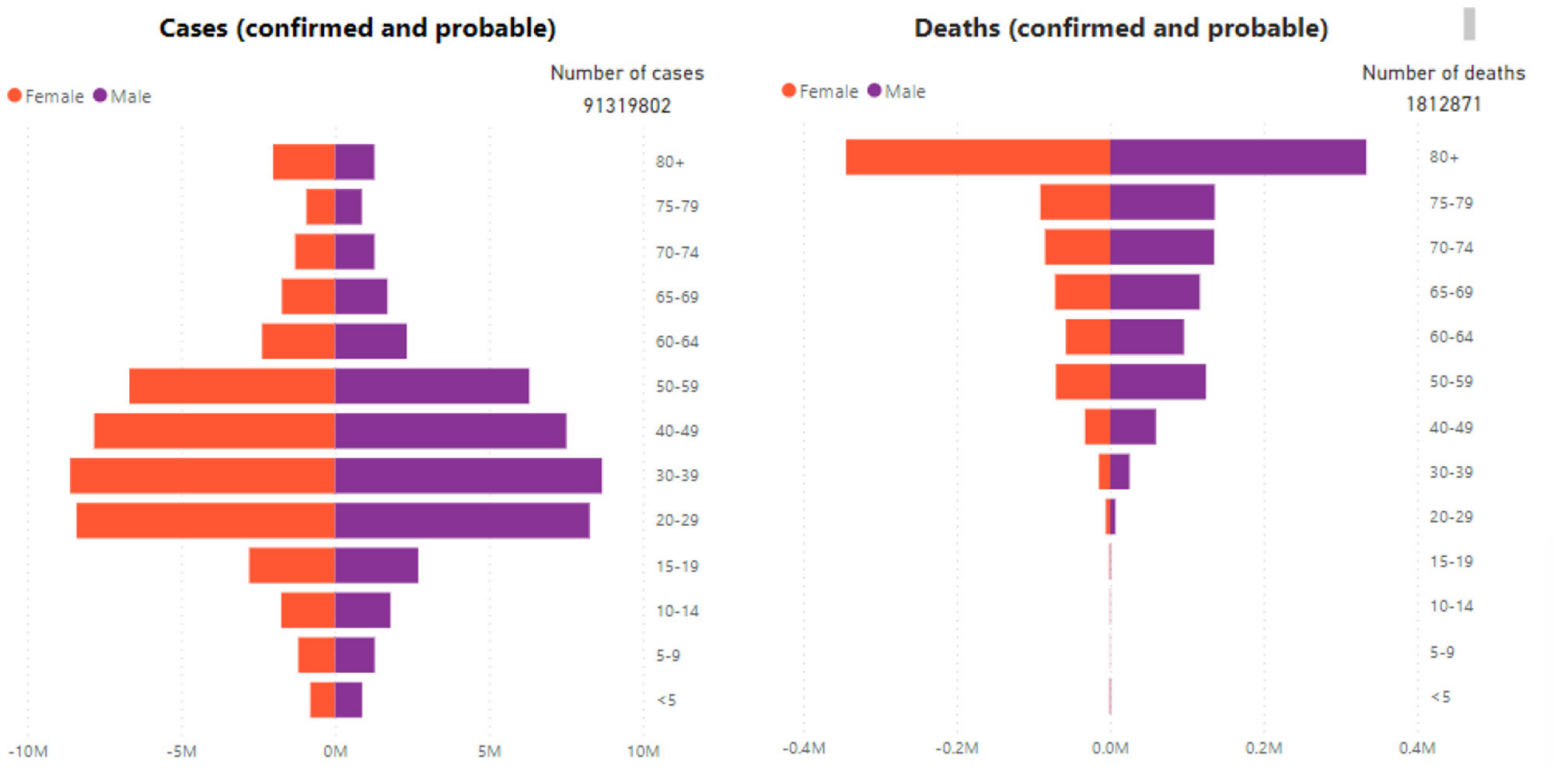
COVID-19 cases and fatalities were reported by age and gender (see text footnote 1).

According to WHO, there were 33,766,707 confirmed cases of COVID-19 in India between January 3, 2020, and October 1, 2021, with 448,339 fatalities. 870,708,636 vaccine doses were delivered as of September 27, 2021^3^ The worldwide COVID-19 cases and fatalities by region, in absolute numbers, as of September 27, 2021, are shown in Table 1 (Dhama et al., 2020)^4^ These regions are adapted according to the World Bank. The worldwide COVID-19 cases and fatalities were reported by age and gender between 30 December 2019 and 27 September 2021, which is summarized in Figure 2 (see text footnote 1).

**Table 1:** COVID-19 cases and fatalities by region (Dhama et al., 2020, see text footnote 4).

**Region**	**Total cases**	**Total deaths**
South America	37,737,608	1,151,181
North America	44,548,923	710,757
European Union and the UK	45,509,611	896,885
Other Europe	12,406,547	179,154
Central America	5,679,357	316,053
Russia and Central Asia	10,044,890	239,175
Middle East	13,169,156	195,989
Caribbean	1,833,102	20,779
South Asia	38,017,617	533,859
Oceania and islands in East Asia	8,661,338	200,334
North Africa	2,498,484	66,730
Sub-Saharan Africa	5,786,014	143,124
East Asia	5,920,734	88,922
Totals	231,813,381	4,742,942

The COVID-19 crisis has created a social, economic, and wellness upheaval. Lockdowns have resulted in people losing their employment. These things have created tremendous pressure on people's mental health and badly affected them physiologically. The facility of online working has helped the industry survive by working from home. However, those industries that have been badly affected involve physical work like labor, technicians, etc. People who belong to poor-class family-like labor, street workers, are badly affected by the lockdowns. The lockdown has enormous negative consequences, particularly for the economy.

In terms of vulnerability, millions of people in India are being dragged into poverty, food insecurity, health, and online education availability issues. From the other perspective, COVID-19 pandemic has given space to many innovations. An Automated System to Limit COVID-19 Using Facial Mask Detection in Smart City Network has been proposed by Rahman et al. (2020). Scalable Telehealth Services to Combat Novel Coronavirus Pandemic was successfully demonstrated by Ullah et al. (2021). Wearable technology to assist patients infected with novel Coronavirus has been implemented by Islam M. et al. (2020). Breathing Aid Devices to Support Novel Coronavirus Infected Patients demonstrated by Islam et al. (2020).

This paper focuses on Computer-aided diagnosis (CAD), which has emerged as a key research topic in medical image processing and clinical diagnostics. Deep neural networks have recently achieved advancements in object identification, semantic segmentation, and picture classification in image recognition tasks. Robust object recognition from medical photos may relieve clinicians of a significant effort, provide solid quantitative assessments, and speed up the diagnosing process.

However, efficiently and successfully detecting sparsely dispersed items from large-scale data remains a difficult task.

- There is significant intra-class heterogeneity in the look of objects in medical photographs.
- Because the items are poorly dispersed, the approach must be efficient and resilient when applied to massive amounts of data.

The upcoming sections of this paper are organized as follows. Section Objective establishes the boundaries of the paper. Section discusses COVID-19 Diagnosis Using Chest Radiographic Images and Deep Learning. Section gives a detailed discussion of the noteworthy literature contributions to detecting COVID-19 infections. Section presents the Overhead for Detecting COVID-19 Using Traditional Methods and Improving the Detection Method Using Deep Learning Techniques. Section presents Recommendations. Section briefs Recommendations for Cost Minimization for Health Services. Section presents the Framework and Research Methodology used in this paper for shortlisting the dataset and manuscripts. Section gives the pointers for future research directions. Section concludes the paper.

## Objective

The paper's motive is to create a comprehensive analysis detection methodology based on Deep learning for COVID-19 diagnosis. The contributions of this paper are three-folds, are summarized in the following points,

- Study and comprehend the noteworthy Deep learning methods for COVID-19 diagnosis.
- To establish the financial Overhead for detecting COVID-19 using traditional methods and improving the detection method using deep learning techniques.
- To establish the Recommendation for Cost minimization for health services.

## COVID-19 Diagnosis Using Chest Radiographic Images and Deep Learning

### Graphical Based Diagnosis Method for COVID-19

The apprehension methods used to distinguish the symptoms, like CT, identify discrete opacities contrasted to the healthy lung image and nucleic acid analysis, which implements real-time multiplex RT-PCR of well-known pathogens and generates negative and positive results (Serte and Demirel, 2021). The immediate preference for COVID- 19 symptomatic research, according to WHO, is the precedence of nuclear acid and protein tests, which effectively detect COVID-19 with the assistance of point-of-care detection. Serological examinations relating to protein are required in joining nucleic acid tests to advance surveillance exercises. This test serves to help with detection after healing and equips clinicians to trace infected and healed patients and to have a more reliable evaluation of an infection. Devices used to evaluate patients, like external periphery devices, can be implemented in areas such as city stations to reduce the load on medication laboratories (Irmak, 2020), where therapeutic care tests are priced optimally, conveniently, and easily handled.

Graphical-based symptomatic detecting techniques using deep learning in pandemics serve an essential purpose in screening for causes that comparatively render better outcomes than traditionally examined CXR and CT scans, amongst the principal radioscopy mechanisms in recognizing and diagnosing COVID-19 virus cases. The deep learning methodology is applied to investigating radiology images (Irmak, 2020). Appropriately identifying the COVID and non-COVID cases is a critical challenge in the non-COVID condition. For example, if people are grieving from bacterial pneumonitis dysfunctioning or other associations of pandemic infection are easily correlated with irregularity COVID-19. Similarly, lung-related diseases such as Pneumonia, which are strange, are infected with COVID but demonstrate similar symptoms, and all the diagnosis methodologies demonstrate the same kind of result.

### Deep Learning Applications for COVID-19

The synthesis of in-depth training with medication and wellness is an innovative path to promote cutting-edge techniques. The paper encourages deep learning study to consider extensive applicability in multidimensions and effectively identify challenges such as COVID-19 or pandemic diagnosis. It is critical to accumulate expertise in crosswise utilization, such as data retrieval, image analysis, or protein structure forecast. It has potential and significant specific application with cutting-edge technology to intensify the supervised learning means for the appropriate diagnosis of Coronavirus. Deep learning has a big impact on the COVID-19 epidemic and opens up new research avenues. Deep learning has applications in simple semantic processing, machine perception, biology, and epidemiology. It broadens the scope of future repercussions, reveals incongruous data patterns, or interprets common sense. Precision diagnosis, protein structure prediction, and therapeutic repurposing are all possible.

Convolutional neural networks demonstrated their potential and established themselves as one of the most prominent deep learning algorithms and powerful techniques for detecting anomalies, irregularities, and diagnostics in chest radiography (Amyar et al., 2020). During a pandemic crisis, researchers focus on analyzing appropriate COVID-19 diagnoses by implementing a Convolutional Neural Network (CNN). Many studies have revealed that using deep learning algorithms could enhance the detection features of CT scan images and the consciousness, specificity, and efficiency of diagnosis (Zhang H. T. et al., 2020). Deep learning technology is a practical, valuable and suitable technique that can be deemed reliable for adequate diagnosis of the COVID-19 virus (Thepade and Jadhav, 2020). It demonstrates the potential to enhance image characteristics through artificial intelligence and distinguish the most inexpensive and most trustworthy imaging methods to anticipate dreadful viruses. Various researchers have recently carried out the application of deep learning for COVID-19. Some of the noteworthy contributions in the literature are from Asraf et al. (2020); Islam M.Z. et al. (2020); Muhammad et al. (2020); Islam et al. (2021); Rahman et al. (2021); Saha et al. (2021); Zhao et al. (2021), and Khasawneh et al. (2021).

Although the application of deep learning methods became game changers in identifying the COVID infections from the image data, researchers also come across a hard time efficiently implementing the solutions due to problems like Intra-Class Variation, Scale Variation, View-Point Variation, Occlusion, Illumination, and Background Clutter in the image data set.

## Discussion

Deep Learning is a subset of Machine Learning which can be used to train in supervised, semi-supervised, and unsupervised models. This is inspired by Artificial Neural Networks. Deep Learning can extract features from the data in a hierarchical fashion, i.e., at first low-level features are extracted, then mid-level features, and finally, high-level features are extracted. The main difference between machine learning and deep learning is that in machine learning, the dataset should be preprocessed thoroughly before being used to train the model. The data preprocessing step is not mandatory in deep learning since the models are robust to noise and missing data. Little to no data preprocessing is required.

Advancements in learning algorithms have given us various deep learning architectures during the last two decades. This helped us expand the number and type of problems that neural networks could solve. Deep learning is not a stand-alone approach, but it is a range of classification algorithms that one can apply to a range of problems.

The upcoming sections discuss the application of the Deep Neural Network in combating COVID-19. The data from the repositories given in Table 2 is used to evaluate and compare the effectiveness of the related works used in this paper.

**Table 2:** Dataset used in different survey papers.

**Dataset classes**	**Dataset used and its link**
Multiclass-COVID19, community-acquired pneumonia, and normal	CT dataset (Ciotti et al., 2020; Jain et al., 2021) Xray dataset [Basu et al., 2020; I.s. of medical and i. r. (sirm), 2020]
Multiclass: COVID19, pneumonia, and normal	X-Ray dataset (Cohen and Morrison, 2020)
Both binary classes–COVID19 and Non-COVID19 and multiclass: COVID19, pneumonia and normal	Xray dataset (Shi et al., 2021, see text footnote 6)
Multiclass	X-Ray dataset (see text footnote 5)
Multiclass	X-Ray dataset (Cohen and Morrison, 2020)
Multiclass	X-Ray dataset (see text footnotes 7, 8)
Multiclass	Xray dataset (Basu et al., 2020; Wang et al., 2020)

**Figure 3:**
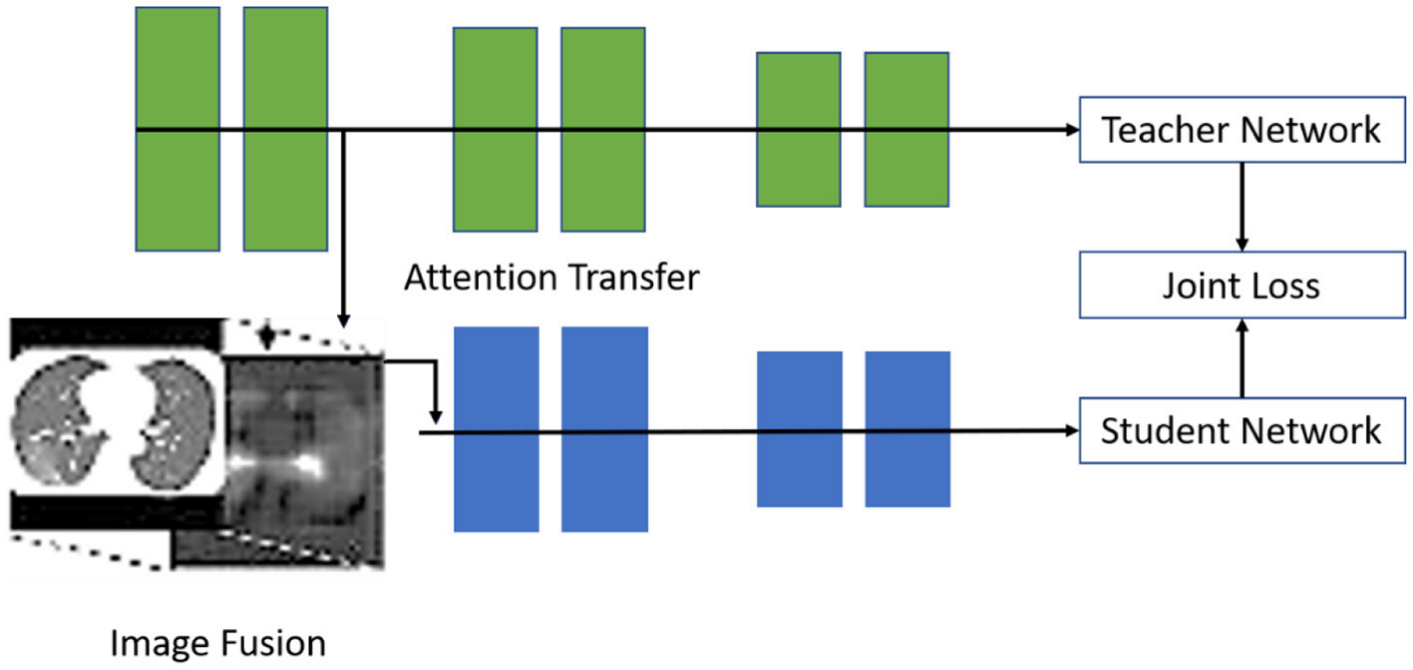
The suggested architecture may be split into a teacher network and a student network (Shah et al., 2021).

The paper (Shah et al., 2021) proposed computer-aided diagnostic (CAD) technology encouraged by artificial intelligence technology to support radiology specialists in examining radiographic images quickly and increasing efficiency by employing the deep learning system and identifying a pattern of COVID-19 features in patients' chest radiographic images. Dataset link [Basu et al., 2020; Ciotti et al., 2020; I.s. of medical and i. r. (sirm), 2020; Jain et al., 2021]. The researchers have developed a knowledge distillation network topology based on explainable AI techniques; the framework of steps used is shown in Figure 3. There are two layers in the network: the teacher and the student network. The attention is generally transferred from the teacher to the student network. The focus of the teacher network is to extract the information that is globally available and emphasizes the critical infection areas to extract attention maps. The deformable attention modules will be employed, if the area of infection is large. This will further help in suppressing the noise in irrelevant areas.

Further, knowledge passed from the teacher's network to the student's network will be used in the image fusion step to combine the original input with the attention information. Normally, the teacher network focuses on the global level features, whereas the student network focuses on learning from the local discriminative features. After this, the concluding experiment is conducted using openly available datasets such as X-Ray/CT Scan images. These experiments established the explainability of the proposed architecture for diagnosing COVID-19.

The proposed methods (Shah et al., 2021) attention mechanism has demonstrated several advantages. Specifically, some state-of-the-art methods for employing attention mechanisms to improve the differentiation power of supervised learning models for X-Ray image processing tasks have been developed. The suggested architecture is shown in Figure 3.

The suggested approach^5^ employs an X-Ray human chest dataset of un-infected individuals, pneumonia-affected and COVID-19 patients. For feature extraction, local dual patterns with mutable input attributes are considered. Many machine learning algorithms and ensembles classify the generated feature sets. Ten-fold cross-validation tests achieve experiment results. To compare performance, various accuracy comparison matrices are used. Results indicate that the Random Tree—Random Forests K-Nearest Neighbor (RTree-RForest-KNN) ensemble provides the best COVID-19 identification method proposed in the paper (see text footnote 5) based on two distinct stages, extraction and classification, where a local binary pattern is applied for extraction, which is further classified by a machine learning algorithm based on a 10-fold cross-validation testing methodology. The outcome illustrates that the R-tree, R-forest, and KNN frameworks achieve the best performance, whereas ensemble models outperform most individual classifiers. When comparing the Local binary patterns (LBP) input parameters, *R* = 6 (*P* = 48) and *R* = 7 (*P* = 56) provide the optimal result for 10-fold cross-validation in this COVID-19 identification method. Dataset link (Cohen and Morrison, 2020; Kim et al., 2022).

**Figure 4:**
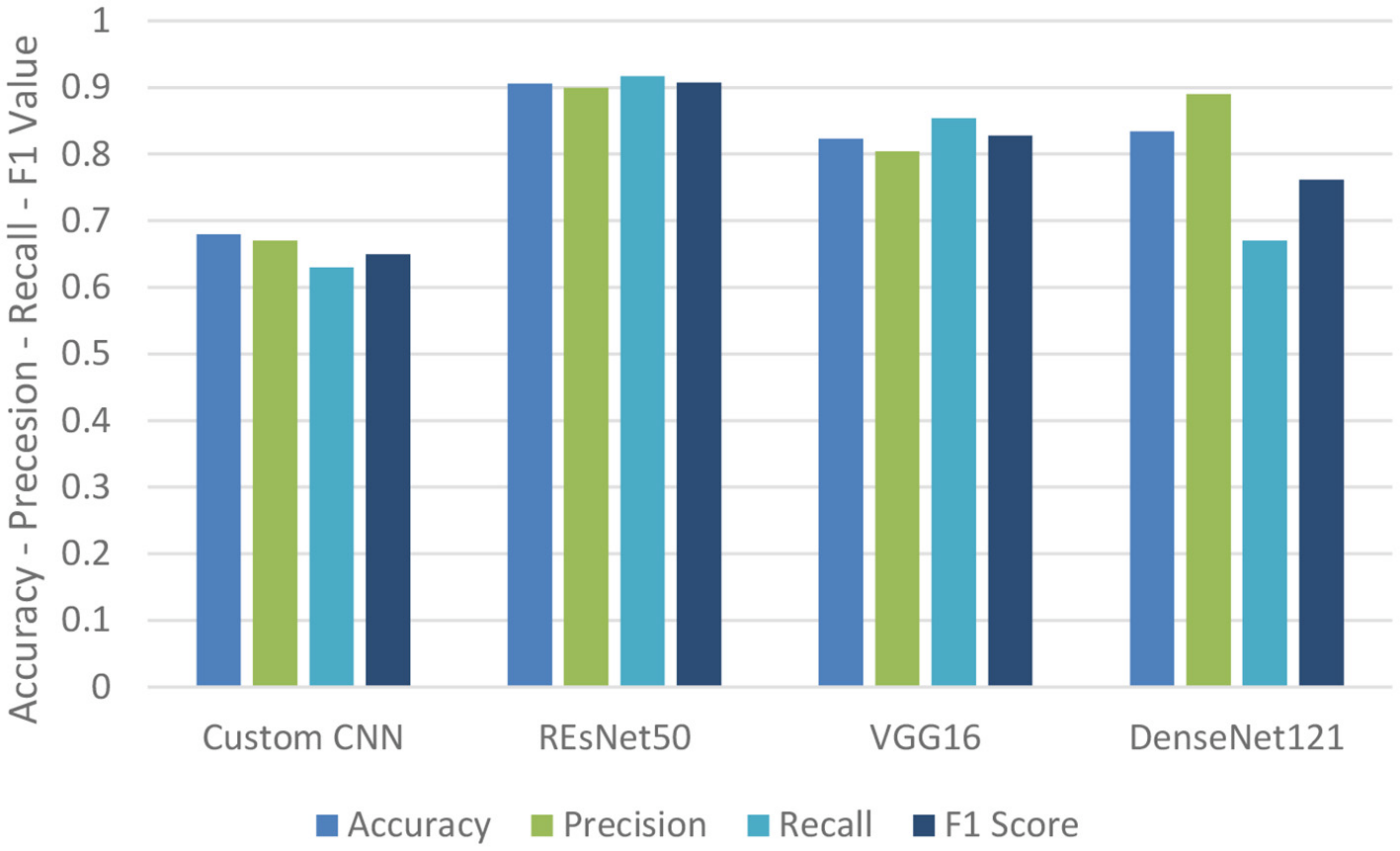
Performance metrics table for binary classification (Haritha et al., 2020a).

The researchers (Haritha et al., 2020a) propose a methodology to locate the region of infection using X-Ray images of the pulmonary area. The authors recommended two strategies because of the limited medical data and computation power available. First, developing a custom convolutional neural network model and training it by using a vast dataset of non-COVID-19 images of chest X-Ray of patients (ChexPert). Finally, tune that custom CNN model with COVID-19 X-Ray images; unfortunately, it did not provide optimal results. Thus, another proposed method used in the paper is transfer learning through a pre-trained CNN model based on Resnet50, VGG16, and Densenet121. The paper reached up to around 90 percent accuracy through the Densenet121 CNN methodology. The performance result is shown in Figure 4. Dataset link (Shi et al., 2021)^6^

In the next step, the Multiclass scenario is considered by extending the problem by considering multiclass classification to identify if the patient is healthy, has Pneumonia not caused by COVID-19, or has COVID-19. The DenseNet architecture was employed for this prediction challenge, and the results metrics are shown in Table 3. The advantages of this paper are that to solve the problem of a lack of COVID-19-related data, the technique of transfer learning was employed, which provides improved accuracy.

**Table 3:** DenseNet121 performance for multiclass (Haritha et al., 2020a).

**Number of images**	**Prediction**		
	**Healthy (%)**	**Pneumonia (%)**	**COVID19 (%)**
12	41.7	33.3	25
24	33.3	45.8	20.8
12	33.3	25	41.7

The researchers (European Centre for Disease Prevention Control, 2020) in the paper concentrate on the detection of COVID-19 using transfer learning. This study uses a dataset of Chest X-Ray comprising 219 images of legitimate COVID-19 chest X-Ray images, as well as 1,341 and 1,345 images of ordinary and pneumonia cases, respectively. The database used for this experimentation is an open-source database available on the Kaggle site. All images are in PNG format and have a 1,024 × 1,024-pixel resolution. This data set has been subjected to many image preprocessing operations.

For this purpose, they used a distinct CNN model to achieve a good result. From the same perspective, the study (European Centre for Disease Prevention Control, 2020) examines pre-trained architectures of deep neural networks, including the ResNet50V2, VGG16, Xception, VGG19, MobileNetV2, and InceptionV3. Finally, they produced the statistical assessment metrics after drawing the confusion matrix. After examining the results, it was revealed that the Xception model produced an effective classification system compared to other models. The outcomes suggested that the Xception network performs significantly among all the models as it attains accuracy and sensitivity of up to 98 and 100%, respectively. All models, on average, attain a high level of precision of 97–98%.

Future research aims to create a model that integrates two transfer learning models to increase performance. This allows for a more accurate and timely diagnosis of COVID-19 patients.

To subdue the transmission of the pandemic in remote rural areas where people cannot afford expensive medical treatment, including the diagnostic expenses of the dreadful disease. The authors of the paper (Qjidaa et al., 2020a) proposed the AI system as a clinical decision support system (CDSS)for rapid and immediate diagnosis of COVID-19 from a chest X-Ray, which illustrates the dynamic potential (Qjidaa et al., 2020a). It is easier and less costly to access X-Ray imaging facilities for people living in rural locations. Five hundred and sixty-six radiology images were collected under three categories: COVID-19, Pneumonia, and Normal. The experimentation was carried out on 30% of test and validation data, and 70% of the original data was used for training. The proposed method is based on transfer learning, which uses seven distinct pre-trained models: VGG16, InceptionResNetV2, VGG19, Xception, DenceNet121, MobileNet, and InceptionV3. After analyzing the results of all models, combine the predicted classes of VGG16, VGG19, IceptionV3, Xception, DenseNet121, InceptionResNetV2, and MobileNet in a vector, and choose the class most frequently predicted by all models. It is possible to build a final classifier with a test accuracy of 99%, f1-score of 98%, precision of 98.60%, and sensitivity of 98.30% by employing this ensemble model methodology (Qjidaa et al., 2020a). This compiled technique demonstrates that the recommended methodology has a high potential to diagnose COVID-19. Dataset used (Cohen and Morrison, 2020).

The suggested model (Ghaderzadeh and Asadi, 2021), with 99.9% accuracy, the proposed model correctly classifies the binary classes of COVID-19 and normal images of patients' chest X-Ray images. CheXNet is a CNN architecture network trained to identify chest X-Ray abnormalities using the ChestXray14 dataset. In general, this model was enhanced to detect all 14 diseases in the dataset of chestXray14, and it employed a pre-trained Densenet121 model to identify COVID-19 and Normal binary class classifications. The dataset used to build this framework incorporates 1,824 evenly distributed thorax X-Ray images of COVID and non-COVID classes, i.e., COVID-19 is confirmed in 912 X-Rays and 912 non-COVID X-Rays. The dataset consists of two sets, training, and testing, with a ratio of 80:20.

The images are initially downscaled to 224 × 224 before being normalized and augmented with horizontal flipping, rotating, zooming, and rescaling, among other things. The initial model of CheXNet made use of the ChestX-Ray14 dataset, which is now the biggest accessible collection of chest X-Rays. CheXNet is a 121-layered deep Neural Network of the CNN type. This network generates a heat map to help with localization. This model was built in this paper (Ghaderzadeh and Asadi, 2021) using the dataset named ChestX-Ray14, which included radiographic images of X-Rays type from 14 distinct pathologies. Four professional radiology specialists classified the test set images in the dataset, and the model's output performance was compared. CheXNet was designed primarily to predict Pneumonia. Training the DenseNet121 model using 1,824 pictures showed outstanding results demonstrating that this model can predict with an accuracy of 99.9%. The improved performance, i.e., accuracy, of this model is attributable to the dataset used—since it represented the distinction—and another factor is that it considered the weights of pre-trained models that have already been trained using chest X-Ray images^7^,^8^

The researcher in the paper (Panwar et al., 2020a) recommends a therapeutic decision assistance method for the initial exposure of a pandemic utilizing chest radiology photographs and a deep learning methodology—the Dataset link (Basu et al., 2020; Wang et al., 2020).

The proposed methodology suggests a clinical decision support system that uses deep learning for the detection of COVID-19 based on chest X-Ray pictures. The architecture is divided into three stages to achieve this objective. The first stage entails input image preprocessing operations and then a data expansion process to increase the data size. The second step includes the extraction of features, followed by learning steps. Finally, a completely connected network with several classifiers is used in the third step to produce the classification and prediction process. This architecture has shown an internal validation area under the ROC Curve (AUC) of 0.97 and an external validation AUC of 0.95. The sensitivity of 0. 92, internal and external validation of 0 and 0.87, with specificity of 0.96 and 0.93, accuracy of 92.5 and 87.5%, negative predictions of 0.97 and 0.93, and 0.92 and F1 scores were 0.92 and 0.88. The outcome demonstrates that the design demands have the potential to distinguish and diagnose COVID-19 adequately to provide for specific, speedy, and practical clinical assistance practice in virus detection

The diagnosis equipment's capabilities are insufficient, creating ambiguity and inconsistencies among patients and physicians over the COVID-19 epidemic. Artificial intelligence exhibits the ability to fix the difficulty through its assistive and adequate usage in COVID exposure and forecast method. The study shows a model of COVID-19 foresight for thorax X-Rays using CoviNet. CoviNet, a deep learning network, was introduced in this paper (Knipe, 2020) to identify COVID-19 in chest X-Ray scans automatically. The foundations of the proposed design are a convolutional neural network, histogram equalization, and an adaptive median filter (Dodds et al., 2020). It is trained using a publicly available dataset. The model had a binary classification accuracy of 98.62% and a multiclass classification accuracy of 95.77%. This framework can aid in radiologists' work in COVID-19 diagnosis.

Many data preprocessing steps are performed, like labeling of dataset images (0 for normal images, 1 for COVID-19 patients' scan images, and 2 for other pulmonary illnesses), keeping images in grayscale, and using data augmentation approaches such as random rotation, vertical flip, and mirroring to solve the

**Figure 5:**
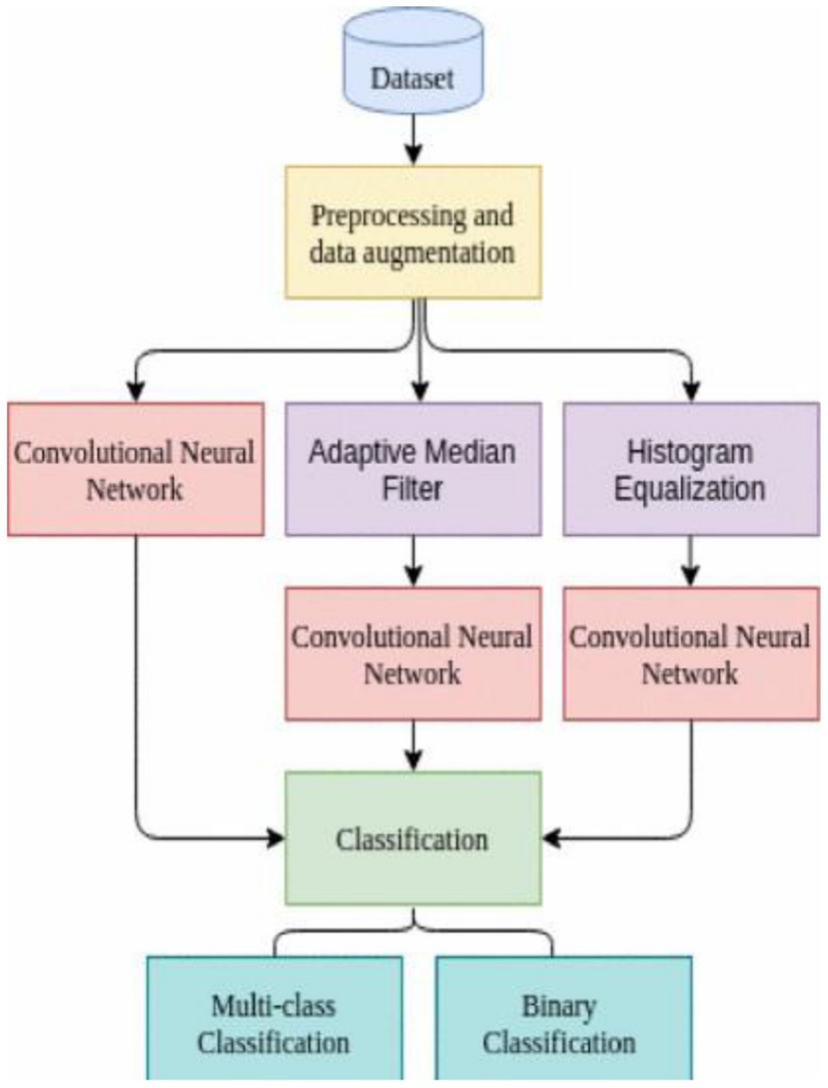
Multi-layered convolutional neural network-based flow diagram of CoviNet (Knipe, 2020).

The problem of data set class imbalances. CoviNet comprises an automated extraction feature and a classification component. CoviNet is based on a multi-layered CNN architecture (Knipe, 2020). After every convolution layer, the rectified Linear activation Unit (ReLU) was used as an activation function. By introducing max-pooling layers, computation complexity is minimized, and the feature maps generated. The resulting pooled output is then flattened and sent into the fully connected layer. The dropout is used to prevent overfitting concerns as a regularization strategy. Larger clinical datasets can be used in future work to properly test the model's performance and improve the segmentation step. The architecture of this model is shown in Figure 5.

The research (Cohen et al., 2020) aims to help radiology specialists interpret radiographs faster and more accurately by offering a deep learning system (Mask R-CNN) to analyze images and discover COVID-19 patterns in patients. In this study, a model based on X-Ray scans was developed to determine the severity of Pneumonia using a bounding box around the infected area.

Attempting to get deeper insights by making research more explainable. These bounding boxes might be used to train a Mask RCNN to classify imagery with Pneumonia and identify where Pneumonia is present within the image. This can assist doctors and radiologists in making more accurate patient diagnoses, saving time, and improving accuracy.

The initial images obtained from the dataset are in Digital Imaging and Communications in Medicine (DICOM) format. These are medical images that are saved in a specific format known as DICOM Files (^*^.dcm). The primary goal of understanding the data structure, image file format, and label kinds is to detect the bounding boxes consisting of binary classification, i.e., presence (or) absence of Pneumonia. The dataset's classifications are split into Pneumonia (lung opacity), no pneumonia (lung opacity—not normal), and normal. Although the dataset is multiclass, a binary classification was employed to detect Pneumonia as positive (or) negative. If positive, what is the ROI for determining the severity of Pneumonia? (Cohen et al., 2020).

The Mask R-CNN X-Ray image can be more specific, reliable, and expertly diagnose the disease by recognizing the severity and the type recovered from the patient's image pattern through deep learning. The paper (Darapaneni et al., 2020) comprehensively analyzes a distinct set of data from Radiological Society of North America (RSNA) X-Ray images, one with Pneumonia and another with no pneumonia but irregular, and the third is the usual type. With the advancement of artificial intelligence, the CNN network recognizes individuals and serves their future potential. This will significantly affect experts focusing on critical cases to avoid the further expansion of the pandemic crisis. The outcome demonstrates that the data set's mean average precision (mAP) is achievable up to 0.90 for train data and 0.89 for test data.

Encouraged by artificial intelligence technology and to support radiology specialists in rendering images quicker and increasing efficiency by employing the deep learning system to examine the image and identify a pattern of COVID-19 in patients. The capability of measuring equipment is inappropriate, which engenders uncertainty and unreliability among patients and physicians regarding the COVID-19 pandemic. The 3-class (COVID-19, Normal, and Viral Pneumonia) detection research (Mohammed et al., 2020) was carried out in this study using SVM, employing.

Feature maps are derived from the ResNet-50 model, a CNN. Each image yielded a 1 × 1,000 attribute map. The research was conducted on 10 repetitions using a 5-fold cross-validation approach.

Residual Network, particularly ResNet 50 has been used to extract the features from the images. Further, the features extracted with the help of ResNet 50 have been fed to Support Vector Machines. Identifying COVID-19 with this method has reported the 96.35% in sensitivity (with the help of 5-fold cross-validation). The classification accuracy with SVM was reported near 99%. Depending on these promising results, it is anticipated that this approach will assist medical professionals while reducing the amount of incorrect identification.

Another work (Qjidaa et al., 2020b) describes CovStacknet's new distribution model based on the StackNet meta-modeling technique. Three datasets are used in the article. The very first dataset (DS1) contains 5,216 X-Ray pictures, 4,273 of which are pneumonia cases and 1,553 of which are normal cases. The second dataset (DS2) was made openly available for experimentation by Kaggle. The third dataset was made available to the public by the University of Montreal. In this work, Convolutional Neural Network named VGG 16 was employed to extract the required features from X-Ray. The feature set consists of 25,088 real array values for every picture, which the StackNet classifier will utilize as features.

The initial technique to feature engineering is a filter-based method in which each feature's variation is calculated, and features with variations smaller than a certain threshold (VarT) are discarded. When a character does not vary considerably within itself, it has minimal predictive value. The second feature selection strategy is feature rank with random forest. This method recommends more essential features to examine throughout the classification process. The Synthetic Minority Over-Sampling Technique (SMOTE) is the best way to handle unbalanced datasets. This strategy oversamples the instances in the minority class in the training dataset using the K closest neighbors' technique. Then, using the flattening layer VGG16, perform tensor flattening modifications to convert the feature matrix into a vector that can be fed into Stacknet (Qjidaa et al., 2020b).

They employed a variety of models based on several techniques to create a robust and efficient model, which was implemented using the Python Scikit-Learn package and pystacknet, an implementation of Stacknet. Initially, it consists of five estimators—one on the initial and final levels, the remaining three on the meta/intermediate level. The model classifies the input data into two categories: one with COVID-19 positive and another with COVID-19 negative. The proposed model was able to achieve an accuracy of 97%. This two-stage technique, which combines stages 1 and 3, results in exceptionally high classification scores compared to only one step, such as stage 2.

The research (Huang L. et al., 2020) demonstrates a new CNN framework for COVID-19 detection. The CNN architecture proposed consists of 12 weighted layers. These 12 layers are classified as two convolutional and fully connected layers. The SoftMax classifier receives an input from the fully connected layer, which generates a 2-dimensional feature vector. The output layer comprises two neurons since this model aims to categorize images into two categories: COVID-19 positive or negative. Dataset consists of a total of 625 pictures used in this study. This dataset is further divided into a subset of training, testing, and validation parts. The training part was used for building the model. Testing was used for testing the accuracy of the built model. The validation accuracy was used to find out the loss of the model and its accuracy. This model, which has a 99.20% accuracy, is used to evaluate whether a certain chest X-Ray image of a patient has COVID-19 or not. Experimental findings on clinical datasets validate the effectiveness of the proposed model.

The active and authentic pandemic diagnosis is probably implemented by a CT scan of the infected lung images. By implementing the deep learning methodology, convolutional neural network, the paper (see text footnote 1) is intended for CTnet-10. This model reported the COVID-19 detection with an accuracy of 82.1 percent. In addition, DenseNet-169, VGG-16, ResNet-50, InceptionV3, and VGG-19 were assessed. The VGG-19 outperformed all other deep learning models, with an accuracy of 94.52%. Finally, they concluded that clinicians might use CT scan pictures for automated COVID-19 diagnosis as a quick and effective strategy for COVID-19 screening (Huang L. et al., 2020).

The findings presented in the study (Udugama et al., 2020) demonstrated the use of transfer learning to detect COVID-19 using chest X-Ray images as input data. This method can classify the given input image data into infected and non-infected categories. This method specifically employed joint learning. The algorithms which perform well when joined are chosen, trained, and deployed as transfer learning. The model's training starts only when the input parameters of both the chosen models are feezed. The models which are pre-trained can be of various combinations. The combination of the model is decided and finalized based on the accuracy and loss value. The chosen models are made sure to change their weights dynamically to learn the patterns in the underlying data. The proposed method by the author has reported an accuracy of 96.1%. This method is proved to be effective in initial diagnostic and screening techniques for COVID-19.

The idea of a weakly supervised deep learning model was proposed by Lafraxo and El Ansari (2021). The proposed method by the authors collects the spatial, axial, and temporal information from CT scans. The Long Short-Term Memory (LSTM) is used for this feature extraction. The various resampling techniques are employed to handle the problem of class imbalance. The data is preprocessed with the help of stochastic and tone image mapping techniques. Finally, the suggested framework's performance is assessed using various module combinations. The proposed technique performed successfully in all evaluation measures and experimental scenarios regarding volume level prediction (Lafraxo and El Ansari, 2021). However, various experimental scenarios yielded varied results for slice-level prediction. In general, the integration of slice attention allows radiologists to concentrate solely on the most important parts of the entire CT volume. From a clinical standpoint, the suggested framework can help radiologists predict COVID-19. Furthermore, it lays the door for future studies aimed at detecting COVID-19 from limited and poorly labeled data.

The study proposed by Rabbah et al. (2020) uses an automated method for detecting COVID-19. It takes CT Scan images as input. As a preprocessing step, it uses pre-defined image processing algorithms. These image processing algorithms help to get the exact portion of the lung. The rest of the portion of the image which doesn't show significant information gets discarded. These preprocessing steps help in errors in classification. The algorithms proposed by the authors have significantly improved the accuracy in detecting COVID-19 from CT Scan images. This work mainly uses a feature pyramid network designed for special categories of classification tasks. This preprocessing will allow the model to investigate different picture resolutions without losing data from microscopic objects. Because COVID-19 infections arise in numerous sizes, many of which are tiny, this technique dramatically increases classification performance.

After completing these two steps, the system assesses the patient's state using a specified threshold. First, this system will be tested on Xception, ResNet50V2, and the proposed model. An accuracy of 98.49% has been achieved in classification concerning a single picture. The dataset consisting of 7,996 images was used for constructing, testing, and validating the model built as a part of this research.

Researchers (Dodds et al., 2020) proposed an automated distribution segmentation mechanism for assisting diagnose COVID-19 chest CT scans. This paper presented an original deep learning-based algorithm to identify COVID-19 infection. This work further extended to segment COVID-19 abnormalities with the help of CT scans. Three learning tasks are done concurrently with diverse datasets: segmentation, classification, and reconstruction. A common encoder with three jobs for disentangled feature representation employed a decoder, and for reconstruction and segmentation, it was implemented with the help of a multilayer perceptron. The evaluation of the proposed algorithm was done on the data set with 1,369 patient lung images, 449 of whom have COVID-19, 425 of whom are normal, 98 of who have lung cancer, and 397 of whom have other diseases (Dodds et al., 2020). The results show that this method works exceptionally well, along with a dice coefficient >0.88 regarding the segmentation phase and a ROC of 97% for classification.

The study conducted by Ni et al. (2020) employed a special classification technique maned binary image. This binary image classification technique is powerful and can efficiently distinguish/classify between COVID and non-COVID chest images. Furthermore, the study demonstrates that those who do not test positive for COVID-19 may have Pneumonia or other respiratory disorders. Many tests analyzed CXR and CT-Scan chest pictures to identify COVID-19 patients. The proposed methodology detects COVID-19 cases with 95.61% accuracy, significantly quicker than the traditional RT-PCR testing procedure.

The weights gained from training the proposed model during CT Scan image processing also offer a substantial response to CXR pictures. They also used a color visualization method in this. To detect COVID-19 instances effectively and with greater recall, it is recommended that the model be trained on radiological images of patients with Pneumonia symptoms. This will assist in the diagnosis of pneumonia patients as True Negative. As a result, COVID-19 symptoms are detected in an unbiased manner in real-time (Ni et al., 2020).

The work presented by Hernandez et al. (2020) has employed the automated deep learning algorithm on CT SCAN images. This method has the advantage of quantitatively characterizing the unusual patterns in the lung images. This study collected a rich database. This study evaluated the patient with COVID infection between January 1 and February 3. The results presented in this study are categorized into severe, moderate, and mild infections. The patients are asked to follow up during treatment. The pre- and post-COVID chest scans of the patients are collected and reanalyzed. It was revealed that the chest scan during pre, during, and after COVID has exhibited phenomenally different patterns.

In this study (Hernandez et al., 2020), researchers assessed the continuous changes in pneumonia seriousness in diverse clinical kinds of COVID-19 at reference line and addition scans using a measurable attribute repeatedly generated by a deep learning method using chest CT images. The primary research outcomes were that this deep learning could detect lung changes in COVID-19 infected patients with varying clinical severity. Patients with mild COVID-19 had a shorter time between the onset of symptoms and the first CT scan, indicating that they may have presented at an earlier stage of the illness. The decreased whole-lung and per-lobe QCT-PLO at baseline CT corroborated this. All severe and critical patients had a <90% pulse oxygen saturation. More than 50% of those polled felt dyspnea, which corresponds to a higher proportion of pulmonary opacification as evaluated by deep learning technology. The opacification rate increased considerably in the first follow-up, according to the findings. Nonetheless, there was no considerable rise in opacification % between the first and second follow-ups.

The method of detecting COVID at the image and scan level was proposed by Rahimzadeh et al. (2021). The algorithm's capability on the image level was evaluated first. This method has exhibited the highest classification accuracy level when the CT scan's central images are used as input data. Subsequently, the accuracy of the proposed method for detecting COVID-19 at the scan level is evaluated. It is noted that the percentage of accuracy is improved as the number/size of images in the dataset starts to grow. The proposed model exhibited an AUC of 0.9 and 0.67 for different deep learning architectures.

In this study (Yang et al., 2020), chest CT is crucial for COVID-19 diagnosis because it enables exact quantification and localization of abnormalities. They deployed deep learning-based software to assist in identifying, localizing, and quantifying COVID-19 Pneumonia. COVID-19 infection quantification and assessment by uAI: The uAI Intelligent Assistant Analysis System, a deep learning-based program, was developed by United Imaging Medical Technology Company Limited (Shanghai, China) specifically for COVID-19 evaluation. This artificial intelligence software uses a modified 3D convolution neural network and a combined V-Net with bottleneck components. According to the generalized linear mixed model, the dorsal region of the right lower lobe was the favored place for COVID-19 Pneumonia. A chest CT scan combined with analysis by the uAI Intelligent Assistant Analysis System may successfully detect Pneumonia in COVID-19 patients.

The quantitative determination performance of a deep learning model with radiological specialists in detecting abnormalities in chest CT images from COVID-19 patients is compared in the proposed study (Narin, 2020). A deep learning system for lesion recognition, segmentation, and localization was trained and validated in 14,435 patients with chest CT images and verified infection diagnosis. The quantitative identification performance of the proposed model was compared to the reading reports of three radiological residents and two experienced radiologists as the reference standard, with the accuracy, sensitivity, specificity, and F1 score assessed. The table of comparison between different COVID-19 detection methods and their strengths and weaknesses is shown in Table 4.

**Table 4:** Comparison of various models on COVID-19.

**Title**	**Detection method used**	**Strengths**	**Scope for improvements**
Performance evaluation of transfer learning technique for automatic detection of patients with COVID-19 on X-Ray Images	CNN based model having 6 layers VGG16, VGG19, Inception V3, Xception, ResNet-50V2, and MoileNet V2	Accuracy 98% and Sensitivity 100%	Accuracy and the sensitivity of the model decreases even if the slight increase in the noise level of the input data.
COVID-19 detection through X-Ray chest images	CNN model based on Resnet, VGG and Densenet	Accuracy 90% by Densenet	This model performance parameters dampen with the increasing size of the dataset.
Early detection of COVID19 by deep learning transfer Model for populations in isolated rural areas	CNN model based on Resnet, VGG, Xceptions, MobileNet, DenceNet121	Accuracy 99% Sensitivity 98.3%	Collection of the dataset from the rural COVID19 infected areas is a challenging task.
CoviNet: automated COVID-19 detection from X-rays using deep learning techniques	CoviNet embedded adaptive median filter, histogram equalization, and convolutional neural network	Accuracy 98.6% for the binary group and reveals 95.77% for the multiple class group.	CoviNet model is succumb to class imbalance problem if highly imbalanced data is used for training the classification model
COVID detection from chest X-Rays with deep learning: CheXNet	CheXNet	Accuracy 99.9%	More evaluation of the proposed method can be conducted by increasing the number of hidden layers in the proposed model.
A new classification model based on stacknet and deep learning for fast detection of COVID-19 through X rays images	CovStacknet based on StackNet meta-modeling	Accuracy 98%	
A novel deep convolutional neural network model for COVID-19 disease detection	Two convolutional layers followed by ReLU and max-pooling layers	Accuracy 99.20%	Evaluating the proposed method by employing a range of activation functions is necessary.
Machine learning-based approaches for detecting COVID-19 using clinical text data	K nearest neighbor classifier (k-NN)	Accuracy 96%	The proposed method takes clinical text data. It may contain noise. Noise can result in wrong diagnostic decisions.
COVIDiagnosis-Net: deep bayes SqueezeNet based diagnostic of the coronavirus disease 2019 (Ucar and Korkmaz, 2020)	COVID/Pneumonia/normal (3-class)	Binary: 98.08%, Multiclass: 87.02%	The model uses offline methods to do the preprocessing of the images.
COVID-Net: a tailored deep convolutional neural network design for detection of COVID-19 cases from chest X-ray images (Wang and Wong, 2020)	COVID/Pneumonia/normal (3-class)	Multiclass: 91.3%	Some of the hyperparameters which can be tuned are size of the network, number of layers, dropout rate, learning rate, kernel size etc. By varying these parameters after the initial learning, the performance can be improved over time, which is evident from the COVID-Net.
CovXNet: A multi-dilation convolutional neural network for automatic COVID-19 and other pneumonia detection from chest X-ray images with transferable multi-receptive feature optimization (Mahmud et al., 2020)	COVID/Non-COVID (Binary) COVID/Non-COVID/Pneumonia (Multiclass)	Multiclass: 90.2%	The learning efficiency of the meta layer can be further improved by increasing the gradient of the activation function to obtain the better results.
Application of deep learning for fast detection of COVID-19 in X-rays using nCOVnet (Panwar et al., 2020b)	COVID/Non-COVID (Binary)	Binary: 97.08%, Multiclass: 87.33%	The response rate of the algorithm can be improved by training with a varied range of input images while at the training time.
Automated deep transfer learning-based approach for detection of COVID-19 infection in chest X-rays (Das et al., 2020)	COVID/Pneumonia/normal(3-class)	Binary: 99.02%	Combining multiple transfer learning approaches to the proposed method and evaluating the multiple combined transfer learning method needs to be studied.

The study presented by Majeed and Hwang (2022) sheds light on the role of artificial intelligence (AI) and other latest technologies that were employed to fight COVID-19. This study gives an excellent comprehension of technologies assisted the early detection/diagnosis, trends analysis, intervention planning, healthcare burden forecasting, comorbidity analysis, and mitigation and control. On a broader note, this study has given the landscape of AI innovative applications in effectively combating with COVID-19 pandemic situation.

The medical fraternity and government agencies from various countries have invested huge amounts of revenue and time to invent a medication that can successfully suppress COVID-19. In this appalling time, efforts were made to use Artificial Intelligence and deep learning methodologies to combat the non-medical aspects such as tracking and forecasting outbreaks, detecting the non-compliance of infected patients, containing the outbreak, and identification of the hot zones which will help to contain the infection spread. Deep learning and AI played a pivotal role in detecting, classifying, and swiftly identifying the infection even in the medical image analysis of the dataset, such as CT, MRI, and X-Ray images. In healthcare, the role of AI and deep learning was quintessential and helped medical institutions and hospitals systematically provide faster diagnoses.

Due to the widespread essence of the Coronavirus, patients are being admitted to health care in batches. This had pushed the government and medical agencies to their edge to accommodate the higher number of admissions to the medical facility. Setting up an atmosphere where the patient can get quick treatment swiftly is daunting. Rapid diagnosis is quintessential and proven to be effective in containing the widespread COVID-19 virus. The mortality rate keeps rising worldwide, and it is evident when WHO (World Health Organization) decided to put nCoV as an epidemic disease on February 11, 2020, coiling the term COVID-19 which stands for Coronavirus Disease 2019.

Deep learning techniques also helped combat the socio-economic problems that took birth due to the COVID-19 pandemic. Many countries are using tools such as Dashboards, which are still helpful for the common people to get information about the precautions to be taken, the infection rate, fatality rate, etc. These dashboards are internally using various AI and deep learning models for processes such as data gathering or retrieval, assimilation of data, identification of the valuable insights from the gathered data from the various sources, and providing meaningful insights based on it in the dashboards.

## Overhead for Detecting COVID-19 Using Traditional Methods, and Improving the Detection Method Using Deep Learning Techniques

“Coronavirus disease 2019 (COVID-19) is a highly infectious disease caused by severe acute respiratory syndrome coronavirus 2” (Trehan, 2021).

“While the RT-PCR test is the gold standard for diagnosing COVID-19, it has limiting aspects with certain features that make it difficult to diagnose the disease. RT-PCR is a very time-consuming, complex, costly, and manual process. One of the drawbacks of this method is the need for a laboratory kit, the provision of which is difficult or even impossible for many countries during crises and epidemics. Like all diagnostic and laboratory methods in healthcare systems, this method is not error-free and is biased. It requires an expert laboratory technician to sample the nasal and throat mucosa, which is a painful method, and this is why many people refuse to undergo nasal swap sampling. More importantly, many studies indicated the low sensitivity of the RT-PCR test; several studies have reported the sensitivity of this diagnostic method to be 30–60%, indicating a decrease in the accuracy of the diagnosis of COVID-19 in many cases. Some studies also pointed to its false-negative rate and contradictory results” [PubMed Central (PMC), 2021]. The drawbacks of manual testing include the sparse availability of testing kits and costly and inefficient blood tests; a blood test takes around 5–6 h to generate the result (Trehan, 2021).

“One of the main features of deep neural networks, in terms of their efficacy, is their employed architecture. Deep neural network architectures demonstrate an extraordinary ability to perform various functions for different data types [PubMed Central (PMC), 2021].” According to the findings, deep learning-based models have an extraordinary capacity to provide an accurate and efficient system for detecting and diagnosing COVID-19 cases. Their use in the processing of modalities would result in a significant increase in sensitivity and specificity value.

There are two important aspects of the examined method contributing to the detection and diagnosis of COVID-19 which are the test's duration and cost. Table 5 shows that the duration of the COVID-19 test depends on the used method. In case RT-PCR was detected in ~4 h; while the diagnosis time of CT scans is 21.5 min (see text footnote 1); and in the case of using X-ray chest images, the duration was <5 min (see text footnote 3). The evaluation period includes all processes, such as taking the swap or the image and sending it to the radiologist and relevant doctor. On the other hand, CT-scan and X-ray cost elements only count for imaging. The promoted method aims to support radiology specialists and doctors by automating the process by highlighting important medical features embedded in the medical images and for early identification of possible COVID infection status. This method will reduce the evaluation times and costs compared to manual processes.

**Table 5:** Comparison of detection type and time in AED and INR.

**Method**	**Type**	**COST (AED)**	**Cost (INR)**	**Time**
CXR image	X-ray	220^9^	280^10^	~5 min (Haritha et al., 2020b)
CT-scan image	PLAIN	2420 (see text footnote 9)	5720^11^	~21.5 min (Huang Z. et al., 2020)
CT-scan image	With Contrast	2680 (see text footnote 9)	5720 (see text footnote 11)	~21.5 min (Huang Z. et al., 2020)
RT-PCR	RT-PCR	150^12^	400^13^	~4 h (Won et al., 2020)
The deep learning-based system	Graphical-design-based symptomatic techniques	220	100	~1 min

## Recommendations

The RT-PCR persisted as the most extensively accepted affirmative test for COVID-19 infection. However, this laboratory analysis has various loopholes. The RT-PCR technique on nasopharyngeal and thoracic swabs has low sensitivity and reliability concerns. Consequently, additional designs that couple radiological images, CT and X-Ray clinical indications, and laboratory inspections are practiced to acquire reliable outcomes. The chest CT scan and X-Ray images have been described as having high efficiency.

Artificial intelligence manifests immense potential in the contemporary cutting-edge world. The utilization of deep learning and machine learning techniques on CT scans and X-Ray images has promoted the more specific determination of Coronavirus. Based on chest radiographic images, deep learning and machine learning approaches have high precision in comparing COVID-19 with non-COVID-19 Pneumonia. This technique has expedited the computerized estimation and assessment of these images. To effectively analyze the inevitable role of artificial intelligence in today's medical world, where it has a dynamic capacity to rapidly interpret and diagnose the COVID-19 infection and aid in studying infection sequences.

The paper suggested that an AI-based methodology, like deep learning, is a machine learning procedure that prepares and trains radiographic data for the machine. This information can be bifurcated into corona-affected and non-COVID-19 related Pneumonia. The prognosis methodology for coronavirus detection and its outcomes with high certainty utilizes cutting-edge technology and manifest potential and is extensively practiced in therapeutic image processing and infection investigation studies using deep learning techniques.

Deep learning algorithms face difficulties because of the lack of transparency and representativeness of features in some radiographic images. It is challenging to discriminate between the many imaging features that have been used to evaluate the results. As no particular tactics can detect all lunglike dysfunctions based on just imaging exhibition on thorax CT scan or X-Ray, the relevance of a multidisciplinary strategy is suggested for overwhelming symptomatic effects. Other challenges faced during diagnosis include statutory insufficiency and the availability of enormous learning data. Massive, noisy data-limited recognition of the cross-crossing between computer science and medication, data secrecy, and protection concerns. However, the efficiency of the deep learning design and image-based modules that drive the variance among Coronavirus and other kinds of Pneumonia is being interpreted.

## Recommendation for Cost Minimization for Health Service

Pandemic has changed the working of the majority of organizations. All organizations are moving toward digitizing their day-to-day operations. The insurance industry is not an exception. More and more financial service providers are increasingly moving toward digitization and utilizing AI-based tools. With the emerging wave of Machine Learning, Deep Learning, and Artificial Neural Networks—AI has made its legitimacy and its initial steps in solving the majority of the challenges in financial tech companies. Financial organizations are forced to shift from detecting and repairing work to predicting and preventing mode in this makeshift. This mode of operation needs to be coped up with everyone directly and indirectly associated with the organization.

The ongoing deep penetration of AI technologies is forcing insurance organizations to change according to the wave and cope with the change by adopting it.

### State of the Insurance by 2030

There will be a tremendous change in the operation of the insurance industry in another decade. The currently followed processes in the organizations will be outdated. Advanced technologies will soon replace the legacy systems of operation. This will bring a dramatic change in the insurance value chain.

### Distribution

Insurance distribution will happen much more swiftly without more involvement and contact between the insurer and customer (Kaesler et al., 2020). AI-based algorithms will gather the insurance risk-related policies and the necessary information to make informed decisions. Some of the leading insurance agencies are already testing the distribution change trend.

The insurance agencies will start using an immutable transactional distributed database for record-keeping—the technology called Blockchain. The information present in the blockchain network is known to everyone, but no one can modify it without all participating nodes' consensus. Insurance information that is currently centralized will become public and more transparency will be induced.

### Claims

The primary efficiency of any insurance organization is the fastness and accuracy with which the claim is being processed. The claim processing will remain the paramount factor of importance by 2030. Highly sophisticated algorithms will be employed to claim routing, efficiency, and accuracy in claim processing. Additionally, automatic customer applications will be deployed at the customer's disposal, which can be accessed through mobile, web, or wearable smart devices. Claim triggers and repair services will be automatically triggered upon loss (Fong et al., 2020).

## Framework and Research Methodology

The following Table 6 summarizes the survey framework used for the study.

**Table 6:** Survey framework.

Objectives	1. Literature collection on the recent works done for COVID-19 detection 2. Literature survey on the dataset discovery and aggregation 31. Explore the CNN architectures, machine learning algorithms for the techniques for COVID-19 detection and classification 4. Explore the architectural designs and patterns for basing the proposed work
Survey methods	Systematic exploration of articles, white papers, journals, and medical databases/journals
Databases explored	IEEE-Xplore, Elsevier Journals, Radiopedia.org, Springer databases, acs.org, GitHub databases
Datasets accumulated	CT Dataset (Ciotti et al., 2020; Jain et al., 2021) Xray Dataset [Basu et al., 2020; I.s. of medical and i. r. (sirm), 2020] X-Ray Dataset (Cohen et al., 2020) Xray Dataset (Shi et al., 2021, see text footnote 6) X-Ray Dataset (see text footnote 6) X-Ray Dataset (Cohen and Morrison, 2020) X-Ray Dataset (see text footnotes 7, 8) Xray Dataset (Basu et al., 2020; Wang et al., 2020, 2017; Zhang J. et al., 2020)
Keywords	Deep learning for COVID-19, COVID-19 classification and detection, convolutional neural networks, transfer learning, ensemble learning
Inclusion criteria	COVID-19 classification using CNN's
Exclusion criteria	Machine learning for COVID-19 and its relevant works
#Papers collected	127
#Papers shortlisted	70
Outcomes achieved	1. Architectures summary presented in **Table 4** 2. Datasets examined under the survey is presented in **Table 2** 3. Analysis of each architecture is presented under discussion (Section Discussion)

## Future Research Prospects

In a short span, COVID-19 has posed several challenges and opportunities. Solving these challenges will yield more significant benefits for the research fraternity at large. This section briefs some of the pressing challenges that surfaced due to the pandemic situation. Any researchers willing to take up COVID-19-related research topic can further explore the pointers given in this section. Each one of the pointers given in the rest of the sections can be explored in-depth, and suitable solutions can be proposed.

**Figure 6:**
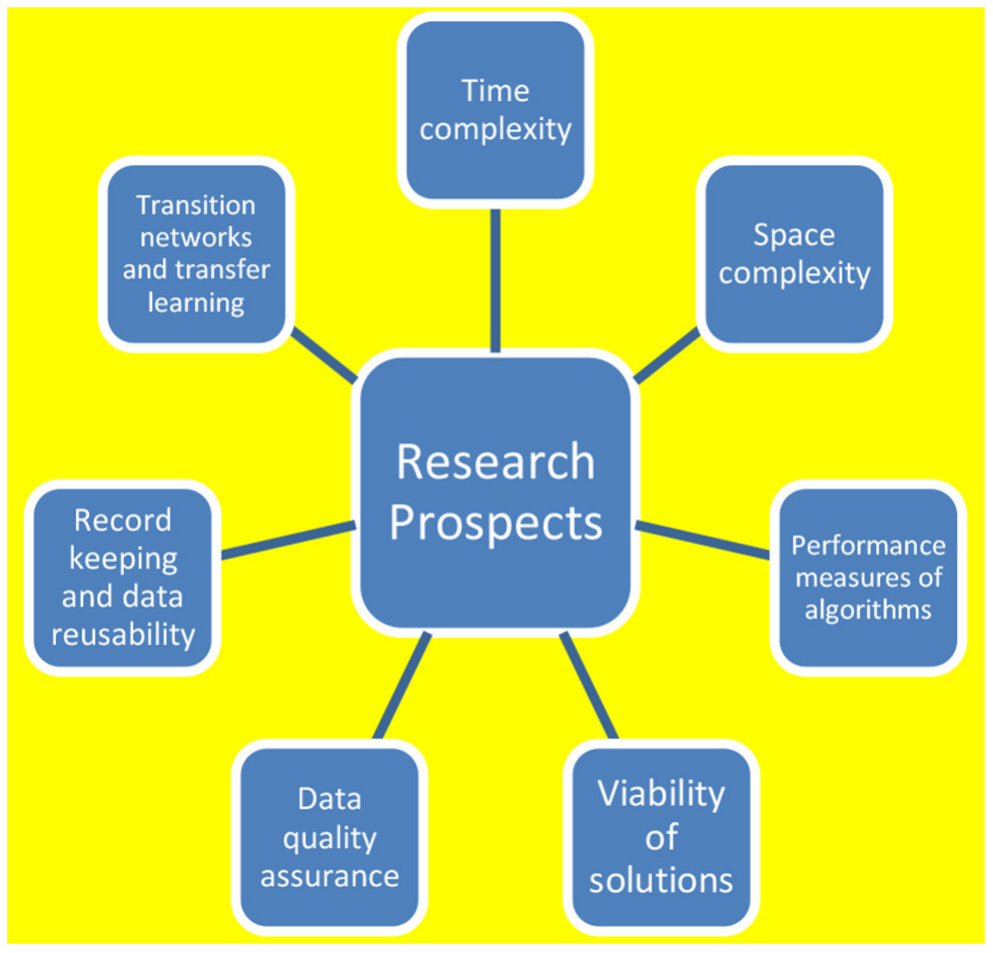
Research prospects.

Data Science related areas could actively contribute to finding out the solutions for threats developed due to infection, severity, and outcome risk. Infection risk is related to groups of people and the most susceptible individuals in that group. Severity risk is related to a specific group of individuals developing severe disease symptoms and complications. Outcome risk is related to the treatments and their outcome. The summary of the future research prospect is illustrated in Figure 6.

There are various realms from which the challenges related to COVID-19 look challenging, if effectively solved, it could serve medical eternity to a larger extent.

The aspects to be considered for enhancement range from time complexity, space complexity, data quality, recording, record keeping, transfer learning, and the aspect of transition networks. All these aspects need to be critically brought under the scrutiny of future research exploration.

There are some other aspects too from which the problems need to be solved. There needs to be methods and techniques that facilitate faster development and discovery of drugs. It is more important to find out if the already available drug can cure the new disease. For this to happen, one should know the ontology of the disease and the characteristics of the new disease. Other dimensions involve understanding the path of the virus, in other words, or to trace the virus entry point. Some other important aspects are Screening patients using face scans. Building biomedical knowledge graphs, predicting drug-target interactions, Predicting the spread of infectious disease using social networks, understanding viruses through proteins, and predicting viral-host protein-protein interactions.

## Conclusion

The application of deep learning in COVID-19 radiologic image processing reduces false positives and negatives and offers a unique opportunity to provide fast, cheap, and safe diagnostic services to patients. The convolutional neural networks have demonstrated their potential and convinced themselves to be among the most prominent deep learning algorithms and powerful techniques in identifying anomalies, irregularities, and diagnostics in chest radiography. During a pandemic crisis, researchers focus on analyzing appropriate COVID-19 diagnoses by implementing CNN technology. The research revealed that using deep learning algorithms could enhance the detection features of X-Ray and CT scan images and the consciousness, accuracy, specificity, and efficiency of the diagnosis.

This paper summarized AI-based deep learning methods and performance challenges to demonstrate rapid virus diagnosis and detection potential. Furthermore, the study allows a comprehensive summary of the existing state-of-the-art techniques and reinforcements for deep learning, CT scan, X-Ray, and machine learning practices exhibited by the more comprehensive wellbeing alliance, expressing how deep learning and machine learning regarding information will heighten the situation of this pandemic.

Additionally, this paper further discusses the cost-effectiveness of the surveyed methods for detecting COVID-19, in contrast with the other methods. Several finance-related aspects of COVID-19 detection and the effectiveness of different methods used for COVID-19 detection have been discussed. This study presents an overview of COVID-19 detection using deep learning methods and their cost-effectiveness and financial implications.

## Author Contributions

All authors listed have made a substantial, direct, and intellectual contribution to the work and approved it for publication.

## Conflict of Interest

The authors declare that the research was conducted in the absence of any commercial or financial relationships that could be construed as a potential conflict of interest.

## Publisher's Note

All claims expressed in this article are solely those of the authors and do not necessarily represent those of their affiliated organizations, or those of the publisher, the editors and the reviewers. Any product that may be evaluated in this article, or claim that may be made by its manufacturer, is not guaranteed or endorsed by the publisher.

## Footnotes

^1^Who coronavirus (COVID-19) dashboard. https://COVID19.who.int/

^2^Who COVID-19 explorer. https://worldhealthorg.shinyapps.io/COVID

^3^India: Who coronavirus disease (COVID-19) dashboard with vaccination data. https://COVID19.who.int/region/searo/country/in

^4^ http://www.sirm.org/category/senza-categoria/COVID-19/

^5^stanfordmlgroup: Xray dataset. Available online at: https://stanfordmlgroup.github.io/competitions/chexpert/

^6^Xray dataset. Available online at: http://www.sirm.org/category/senza~categoria/COVID-19/

^7^ http://md-datasets-public-files-prod.s3.eu-west-1.amazonaws.com/bc9f750d-b663-48a7-844e-4e8246751706

^8^ http://academictorrents.com/details/557481faacd824c83fbf57dcf7b6da9383b3235a

^9^Dubai Radiology Center. Available online at: https://www.drhc.ae/dubai-radiology-center

^10^Cost-of-X-Ray-chest-pa-view-in-India. Available online at: https://www.labsadvisor.com/cost-of-x-ray-chest-pa-view-in-india

^11^CT-scan-cost-in-India. Available online at: https://www.medicoverhospitals.in/articles/ct-scan-cost-in-india

^12^Fakeeh University Hospital—UAE. Available online at: https://fuh.care

^13^COVID-19 Package in Bangalore. Available online at: https://www.apollodiagnostics.in/bangalore/test/covid-19
